# Lessons from an investigation of an autochthonous chikungunya outbreak in Bergerac, mainland France, June to October 2025

**DOI:** 10.2807/1560-7917.ES.2025.30.49.2500871

**Published:** 2025-12-11

**Authors:** Roman Creuse, Alice Herteau, Anne Bernadou, Valérie Cesa, Karim Tararbit, Guillaume Lacour, Delphine Binet, Guillaume André Durand, Florian Franke, Catarina Krug, Laurent Filleul

**Affiliations:** 1 Santé publique France (French National Public Health Agency), Bordeaux, France; 2 Regional Health Agency of Nouvelle-Aquitaine (ARS Nouvelle-Aquitaine), Périgueux, France; 3 Regional Health Agency of Nouvelle-Aquitaine (ARS Nouvelle-Aquitaine), Bordeaux, France; 4 Altopictus, Saint-Jean-d’Illac, France; 5 National Reference Centre for Arboviruses (NRC), National Institute of Health and Medical Research (Inserm) and French Armed Forces Biomedical Research Institute (IRBA), Marseille, France; 6 Santé publique France (French National Public Health Agency), Marseille, France; 7 Santé publique France (French National Public Health Agency), Saint-Maurice, France

**Keywords:** chikungunya, outbreak, local transmission, door-knocking survey

## Abstract

In 2025, an unprecedented number of autochthonous chikungunya transmission events occurred in mainland France. One of the largest outbreaks took place in Bergerac (south-west France). By 27 October, 100 locally acquired cases were identified. They had symptom onsets between June and October. Our data suggest that door-knocking surveys and vector control measures assisted in slowing the virus spread. Autochthonous outbreaks of mosquito-borne diseases are expected to become more frequent with climate change, so improving outbreak control is crucial.

Before 2025, autochthonous transmission events of chikungunya were rare in mainland France. In addition, over the past 10 years, few imported cases have been annually detected [1,2]. From 1 May to 27 October 2025, 1,025 imported cases were identified in mainland France [1], most of these cases had travelled to Réunion Island where a large epidemic occurred [3]. In summer 2025, early and numerous outbreaks of locally acquired chikungunya were reported in mainland France [4]. By 27 October, 17 outbreaks were identified in the Nouvelle-Aquitaine region (south-west France), totalling 154 cases. Before 2025, no autochthonous cases were recorded in the region [1]. Here we describe the chikungunya outbreak in Bergerac, a small town located in Nouvelle-Aquitaine.

## Arbovirus surveillance

In mainland France, chikungunya is a notifiable disease. From 1 May to 30 November, when *Aedes albopictus* mosquitoes are active, surveillance is enhanced to prevent and limit the risk of autochthonous transmission of mosquito-borne pathogens [5]. Public health authorities contact cases by phone to determine whether the infection is imported or autochthonous and to identify places frequented during the viraemic period, i.e. the most probable places of further transmission, and during the exposure period (most probable place of infection). These investigations are conducted according to Santé publique France (SpFrance) protocol and guidance from European Centre for Disease Prevention and Control (ECDC) [5,6].

## Outbreak detection

On 6 August 2025, the Regional Health Agency (ARS) was notified of detection of chikungunya virus (CHIKV) by RT-PCR in a patient (Patient A) living in Bergerac. Patient A had not recently travelled to an area where the virus was known to be circulating, and the onset of their symptoms was on 3 August. On 8 August, the mosquito control operator (MCO) conducted entomological surveys in two potential areas of infection, visited 11 households and identified three suspect cases in the neighbourhood of Patient A (Patients 1, 2 and 3). In addition, the ARS received notification of a second autochthonous case (detected by RT-PCR), Patient B, living in the same neighbourhood but with no other epidemiological link to Patient A. Patient B observed the first symptoms on 31 July. On 11 August, the MCO identified a fourth suspect case (Patient 4) and carried out adulticidal spraying in public areas and 30 private gardens around the residences of patients A and B. Chikungunya was then laboratory-confirmed in Patients 1–4.

## Epidemiological door-knocking survey

On 13 August, eight employees from the ARS and the regional office of SpFrance conducted an epidemiological door-knocking survey within a radius of 150 m of the residences of Patient A and B. This area was considered the most likely location of infection and matched the flying distance of *Ae. albopictus*. The aims of the survey were to (i) identify the primary case and suspect autochthonous cases; (ii) inform residents about the symptoms of chikungunya and what to do if symptoms appear; (iii) offer suspect cases home blood testing and/or a referral to a laboratory for testing; (iv) advise on how to avoid mosquito bites and eliminate mosquito breeding sites in the gardens.

We met residents in 67 (50%) of the 134 households in the area and left information material in the letterboxes of the other households. We identified 11 additional suspect cases: four accepted testing with blotting paper, five had planned or accepted a medical appointment but two children of a suspect case did not consent to testing. Six of these suspect cases were then confirmed. The children’s parent had a negative test result (blotting paper). We could not identify the primary case. Some of the identified suspect cases were living at the boundaries of the first treated area. Therefore, these visits resulted in the MCO extending the area of the second insecticide treatment (carried out on 18 August).

## Case definitions

We adapted our case definitions from the SpFrance surveillance protocol [5] considering the epidemiological context, specifically links to other cases and/or visits to or residence in Bergerac during the exposure period (Box). All suspect cases were encouraged to be tested.

Box **Definitions of chikungunya cases, Bergerac, Nouvelle-Aquitaine region, mainland France, June–October 2025**

**Suspect case:**

• Symptoms (fever and/or arthralgia and/or rash, in the absence of any other infection) between 1 June and 31 October 2025.

**Probable case:**

• Suspect case with IgM antibodies against chikungunya virus (CHIKV).

**Confirmed case:**

• Suspect case with detection of CHIKV by RT-PCR OR IgM and IgG antibodies against CHIKV OR IgG seroconversion on two consecutive samples (at least 10 days).

## Description of cases and outbreak

By 27 October, we identified 11 suspect, 18 probable and 82 confirmed autochthonous cases in Bergerac. Four of the 11 suspect cases were not tested and seven had negative laboratory results. Twelve of the 100 probable and confirmed cases had symptom onsets before the identification of the index case (Patient A) and 88 between 6 August and 3 October. Seven persons were hospitalised (Table). No deaths were recorded. The earliest case presented symptoms on 23 June and the latest on 3 October. Thus, there were 102 days between the symptom onset of the earliest and the latest case, 44 days between the symptom onset of the earliest case and the notification of the index case and 49 days between the earliest case and the first adulticidal spraying. Some cases were tested many weeks after the symptom onset (by which time they sometimes were no longer symptomatic). This is especially true for the earliest case which was identified 60 days after the symptom onset.

**Table:** Characteristics of probable and confirmed autochthonous cases of chikungunya, Bergerac, Nouvelle-Aquitaine region, mainland France, June–October 2025 (n = 100)

Characteristic	n	%
Sex		
Female	57	57.0
Male	43	43.0
Symptom		
Arthralgia	93	93.0
Fever	82	82.0
Rash/exanthema	60	60.0
Headache	22	22.0
Asthenia	17	17.0
Myalgia/back pain	17	17.0
Extremity oedema	7	7.0
Conjunctival hyperaemia	2	2.0
Hospitalisation		
Yes	7	7.0
Age (years)		
Mean (min–max)	60 (7–94)	
Time from symptom onset (days)	Median	IQR
To blood sampling	3	1–7
To notification	8.5	5–14

IQR: interquartile range.

Source: French national arbovirus surveillance system, Santé publique France, 27 October 2025.

**Figure 1:**
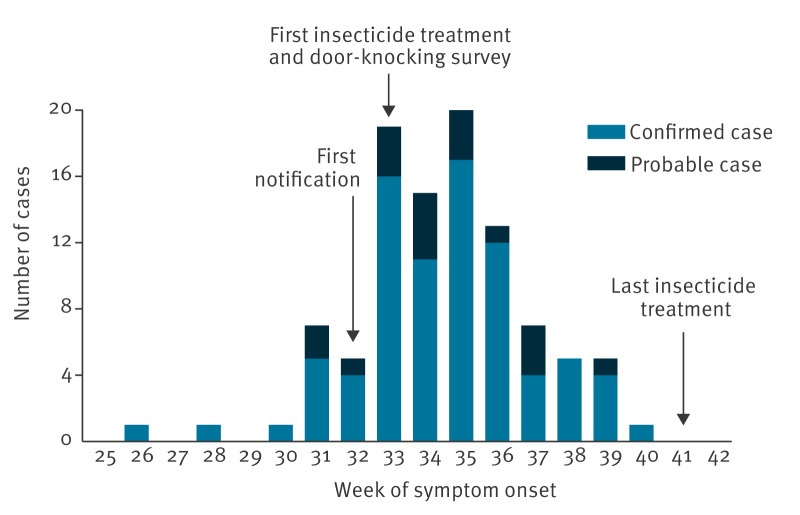
Epidemic curve of probable and confirmed autochthonous cases of chikungunya, by week of symptom onset, Bergerac, Nouvelle-Aquitaine region, mainland France, June–October 2025 (n = 100)

Source: French national arbovirus surveillance system, Santé publique France, 27 October 2025.

**Figure 2:**
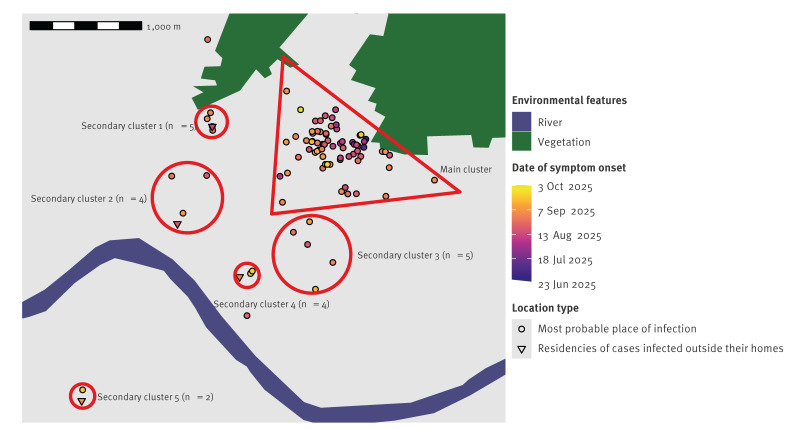
Location of probable places of infection, main transmission cluster and probable secondary clusters in an autochthonous chikungunya outbreak and dates of symptom onset, Bergerac, Nouvelle-Aquitaine region, mainland France, June–October 2025 (n = 100)

Source: French national arbovirus surveillance system, Santé publique France, 27 October 2025.

The outbreak peaked in week 35 (Figure 1). Most (n = 82) cases seemed to have been infected in the same area which we here call the main transmission cluster. However, some cases did not visit this area and were living too far away to have been infected there, given the flying distance of *Ae. albopictus*. A local spread of CHIKV from the main transmission cluster was considered unlikely for these cases. By tracking the movement history of the cases during the exposure period (15 days before symptom onset), we identified five potential secondary transmission clusters (Figure 2). In each secondary transmission cluster, at least one case had a link with the main transmission cluster and then mosquitoes spread CHIKV around their residence. Secondary clusters 1 and 2 were most likely linked to the earliest known case. Cases of the secondary cluster 3 resided around the hospital where several cases from the main transmission cluster were admitted. Secondary cluster 4 was most likely linked to a case working near the main transmission cluster and included three children attending a school in Bergerac but living outside the town. Secondary cluster 5 was most likely linked to parents picking up their children from school in the main transmission cluster. Most (n = 89) cases lived in Bergerac, nine lived in the surrounding municipalities and two in other departments. No other cases were identified in the cities of residency of these cases.

## Laboratory investigations

Clinical microbiology laboratories sent samples with positive RT-PCR results to the National Reference Centre for Arboviruses (NRC) where whole genome sequencing and genotyping were performed. The NRC obtained complete sequences for nine cases and identified the genotype ECSA-2, the lineage responsible for the outbreak in Réunion Island [7]. Sequences from these cases were similar to those from an autochthonous case living in Bordeaux with no history of travel to Bergerac [7]. We could not identify any link between these two transmission events.

## Vector control measures

The MCO applied adulticides within a radius of 300 m of probable places of infection (n = 50 treatments from weeks 33 to 41). In addition, areas frequented by cases during their viraemic period with high risk of further transmission were treated in a radius of 150 m (n = 22 treatments from weeks 33 to 41). The MCO dropped a flyer in the letterboxes of people living close to the sprayed areas to inform them about treatment dates and recommendations until week 39. In weeks 40 and 41, an area of around 460 ha was treated. Residents were informed by text messages and public information. The MCO carried out fewer than 10 anti-larval interventions due to the excessive workload (priority given to treatment preparation and adulticidal spraying).

## Impact of epidemiological door-knocking survey

**Figure 3:**
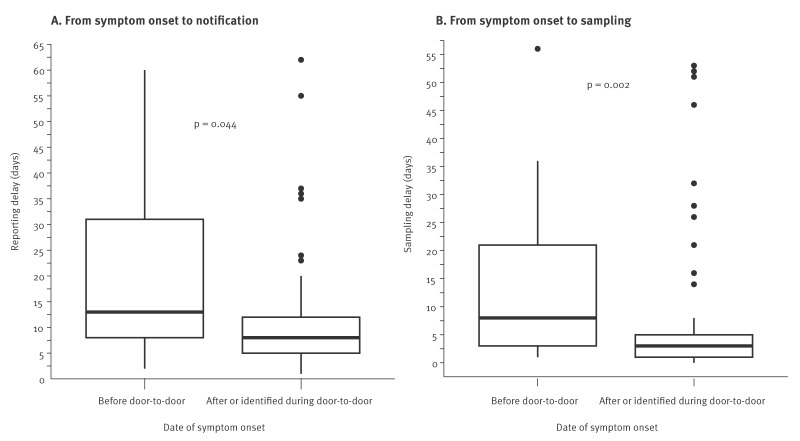
Notification (A) and sampling (B) delay of autochthonous cases of chikungunya with symptom onset before an epidemiological door-knocking survey (n = 17) and cases with symptom onset after or identified during the survey (n = 83), Bergerac, Nouvelle-Aquitaine region, mainland France, June–October 2025

Source: French national arbovirus surveillance system, Santé publique France, 27 October 2025.

The median time from symptom onset to notification decreased from 13 days (interquartile range (IQR): 8–31) to 8 days (IQR: 5–12) between cases with symptom onset before the door-knocking survey and those with symptom onset after or identified during the survey (p = 0.044; Wilcoxon test). Likewise, the median time from symptom onset to sampling decreased from 8 days (IQR: 3–21) to 3 days (IQR: 1–5) between these two groups (p = 0.002; Wilcoxon test) (Figure 3).

## Discussion

An unprecedented number of imported cases of chikungunya were identified in 2025 in mainland France, associated with the outbreak in Réunion Island [1]. In addition, many local transmissions were recorded. Of all the local chikungunya transmission events that occurred in the Nouvelle-Aquitaine region in 2025, the one in Bergerac had the highest number of cases and the longest duration [1]. These local transmission events helped to identify both the strengths and limitations of our current surveillance system.

We conducted entomological and epidemiological door-knocking surveys. By door-knocking, we identified suspect cases which may not otherwise have sought medical care. Only two suspect cases did not consent to testing (children) and people in door-knocked households were willing to discuss mosquitoes and chikungunya. The visits by the MCO were crucial in preparing for the epidemiological door-knocking survey. Similar practices have been applied with success also in other European countries in response to autochthonous vector-borne diseases [8]. We consider that door-knocking also helped in adapting inhabitants’ behaviour and improving their knowledge of preventive measures. However, these surveys were time-consuming and required considerable human resources.

The time from symptom onset to notification varied from 1 to 62 days (60 days for the earliest case). An early identification of the first case is a key factor in outbreak control, also for CHIKV [9], as it enables a rapid response and prompt implementation of control measures. Patients with chikungunya may experience mild and/or unspecific symptoms and thus not seek healthcare which probably contributed to a silent spread of the disease within the community. Furthermore, some healthcare professionals may be unfamiliar with chikungunya considering its recent emergence in mainland France. Delayed identification of autochthonous cases in a 2017 chikungunya outbreak in Italy probably enhanced the spread of the virus [10]. Improving awareness of the disease and the importance of early diagnosis and notification among healthcare professionals and the population may help prevent large clusters in the future [11].

Despite vector control measures, the transmission chain could not be interrupted which resulted in the spread of the virus and many cases, and the dispersion of the MCO efforts due to the need for treatments in many places. However, data collected on the geographic distribution of the cases may have enabled better targeted vector control measures, which may have contributed to slowing the spread of the outbreak.

Schools and hospitals may facilitate the emergence of secondary clusters. Collaborating with these institutions for communication during outbreaks could help prevent such events.

## Conclusion

Outbreaks of mosquito-borne diseases are expected to become more frequent and larger with climate change, the spread of mosquito vectors in mainland France and globalisation. To address this growing challenge, future efforts should therefore focus on improving the knowledge among the healthcare workers and within the community, and the effectiveness of outbreak control strategies, including vector control.

## Data Availability

Data on autochthonous transmission of chikungunya are available on the website of Santé publique France and updated weekly from May to November.
